# Metabolic classification of non-small cell lung cancer patient-derived xenografts by a digital pathology approach: A pilot study

**DOI:** 10.3389/fonc.2023.1070505

**Published:** 2023-02-28

**Authors:** Federica Ferrarini, Elisabetta Zulato, Massimo Moro, Paola Del Bianco, Cristina Borzi, Giovanni Esposito, Tiziana Zanin, Gabriella Sozzi, Stefano Indraccolo

**Affiliations:** ^1^ Immunology and Molecular Oncology Diagnostics Unit, Istituto Oncologico Veneto IOV IRCCS, Padova, Italy; ^2^ Basic and Translational Oncology Unit, Istituto Oncologico Veneto IOV IRCCS, Padova, Italy; ^3^ Tumor Genomics Unit Department of Research, Fondazione IRCCS Istituto Nazionale dei Tumori, Milan, Italy; ^4^ Clinical Research Unit, Istituto Oncologico Veneto IOV IRCCS, Padova, Italy; ^5^ Department of Surgery, Oncology and Gastroenterology, Università degli Studi di Padova, Padova, Italy

**Keywords:** NSCLC, metabolic classification, OXPHOS metabolism, IHC, digital pathology

## Abstract

**Introduction:**

Genetically characterized patient-derived tumor xenografts (PDX) are a valuable resource to understand the biological complexity of cancer and to investigate new therapeutic approaches. Previous studies, however, lack information about metabolic features of PDXs, which may limit testing of metabolism targeting drugs.

**Methods:**

In this pilot study, we investigated by immunohistochemistry (IHC) expression of five essential metabolism-associated markers in a set of lung adenocarcinoma PDX samples previously established and characterized. We exploited digital pathology to quantify expression of the markers and correlated results with tumor cell proliferation, angiogenesis and time of PDX growth in mice.

**Results:**

Our results indicate that the majority of the analyzed PDX models rely on oxidative phosphorylation (OXPHOS) metabolism, either alone or in combination with glucose metabolism. Double IHC enabled us to describe spatial expression of the glycolysis-associated monocarboxylate transporter 4 (MCT4) marker and the OXPHOS-associated glutaminase (GLS) marker. GLS expression was associated with cell proliferation and with expression of liver-kinase B1 (LKB1), a tumor suppressor involved in the regulation of multiple metabolic pathways. Acetyl CoA carboxylase (ACC) was associated with the kinetics of PDX growth.

**Conclusion:**

Albeit limited by the small number of samples and markers analyzed, metabolic classification of existing collections of PDX by this mini panel will be useful to inform pre-clinical testing of metabolism-targeting drugs.

## 1 Introduction

In the last decade, many studies uncovered the metabolic complexity and heterogeneity of cancer. By using advanced technologies such as transcriptomics or metabolomics, distinct metabolic entities have been identified in the main types of human tumors, enabling metabolic classification of human tumors (1–3). Some studies classified tumors based on high, intermediate and low metabolic activity, inferred from expression levels of metabolism-related gene sets (4, 5), whereas others stratified tumors into glycolytic or oxidative metabolism classes according to expression levels of genes/metabolites belonging to glycolysis or oxidative phosphorylation (OXPHOS) (6–8). Recently, a pathway-based classification was proposed in brain tumors based on single cell RNA sequencing, including both mitochondrial and glycolytic/plurimetabolic subtypes endowed with different clinical outcomes (9).

With regard to NSCLC, a landmark study using intraoperative (10)C-glucose infusions in patients compared metabolism between tumors and benign lung and reported marked heterogeneity in tumor metabolism *in vivo*, but also highlighted the strong influence of the microenvironment on this feature (11). Additional studies described lactate uptake and its utilization as a fuel by lung cancer cells (12) and uncovered the role of the microenvironment as determinant of the metabolic phenotype of lung cancer cells *in vivo* (13). Numerous studies have delineated how cell-intrinsic factors, such as oncogenic lesions or epigenetic events, alter cellular metabolism, causing phenotypes characterized by increased glycolysis (10, 14) or other metabolic alterations, as reviewed by B. Majem et al. (15).

When approaching the complexity of cancer metabolism, it can be useful to adopt a simplified classification of tumors into “glycolytic” and “OXPHOS” subsets (6–8). Glycolytic tumors uptake glucose and preferentially metabolize it *via* glycolysis to lactate, which is exported from cells *via* monocarboxylate transporters (MCTs), such as MCT4 (16). In contrast, OXPHOS tumors can utilize several substrates as mitochondrial fuels, the main being represented by glutamine and fatty acids (FA) (17).

In this study, we set-up a panel of immunohistochemistry (IHC) markers including some enzymes and/or transporters belonging to these key metabolic pathways, implementing a panel that we recently used to profile patient-derived xenograft (PDX) samples from ovarian cancer (18). For the purpose of this pilot study, we considered monocarboxylate transporter 4 (MCT4) as proxy of glycolysis (16, 19), acetyl CoA carboxylase (ACC), fatty acids synthase (FAS) and carnitine palmitoyl transferase 1A (CPT1A) as proxy of FA metabolism (20, 21) and glutaminase (GLS) as proxy of glutamine metabolism (22).

We exploited this panel to investigate by IHC expression of these essential metabolism-associated markers in a set of previously established lung adenocarcinoma PDX samples. We exploited digital pathology to quantify expression of the markers and correlated results with tumor cell proliferation and angiogenesis, time of PDX growth and with known driver mutations of the cancer cells. Metabolic classification of existing collections of PDX by this mini panel will be useful to inform pre-clinical testing of metabolism-targeting drugs.

## 2 Material and methods

### 2.1 Patient data

Tumor samples were collected as described by Moro et al. (23). Samples of primary non small-cell lung cancer (NSCLC) were obtained from patients undergoing surgical resection, who gave their informed consent after approval from the Internal Review and the Ethics Boards of the Fondazione IRCCS Istituto Nazionale Tumori and all methods were performed in accordance with institutional guidelines and regulation and with the declaration of Helsinki. Patient data relevant to this study are reported in **Table 1**.

**Table 1:** Clinical and biological features of the NSCLC patient-derived xenografts (PDXs) utilized in this study.

PDX ID	Clinical features			Biological features		
	Stage	Grade	Time of collection	Histo type	Mutated genes	TT(days)
LT 66	IIIA (T1aN2M0)	G3	Relapse	ADC	*CDKN2A, KRAS, LKB1, TP53*	34
LT 111	IIB (T2bN1M0)	G3	Relapse	ADC	*CDKN2A, CTNNB1, KRAS*, *MET, TP53*	37
LT 128	IIIA (T2bN2M0)	G3	Relapse	ADC	*CDKN2A, KRAS, LKB1, TP53*	55
LT 138	IA (T1aN0M0)	G3	Diagnosis	ADC	*ERBB4, FBXW7, LKB1*	73
LT 141	IIIA (T2aN2M0)	G3	Relapse	ADC	*CTNNB1, KRAS, TP53*	104
LT 215	IV (T2aN2M1)	G3	Relapse	ADC	*KRAS, TP53*	37
LT 220	IIIA (T3N1M0)	G3	Diagnosis	ADC	*-*	32
LT 255	IA (T1aN0M0)	G2	Diagnosis	ADC	*CDKN2A, CTNNB1, KRAS, TP53*	32
LT 265	IV (N/A)	N/A	Diagnosis	ADC	*FLT3, LKB1, TP53*	30
LT 267	IIIA (T1aN2M0)	G3	Diagnosis	ADC	*KRAS, PIK3CA*	37
LT 273	IIIB(N/A)	N/A	Diagnosis	ADC	*KRAS, LKB1, TP53*	34
LT 278	IIIA (TxN0M0)	y	Relapse	ADC	*LKB1*	69
LT 305	IIA (T2bN0M0)	G3	Diagnosis	ADC	*APC, KRAS*	38
LT 323	IIIA (T4N0M0)	G3	Diagnosis	ADC	*NRAS, TP53*	35
LT 431	IIIA (T4N0M0)	G3	Diagnosis	ADC	*-*	90
LT 458	IV (T3N3M1)	G3	Diagnosis	ADC	*KRAS, LKB1*	45
LT 497	IV (T3N3M1)	G3	Diagnosis	ADC	*KRAS, LKB1*	40

ADC, Adenocarcinoma; TT, time to transplantation (TT was defined by the tumor size. Tumors were explanted when their volume exceeded 600 mm^3^), N/A, Not Available; Stage, stage refers to initial diagnosis and follow TNM edition 7^th^; Genes: Adenomatous Polyposis Coli (APC), cyclin dependent kinase inhibitor 2A (CDKN2A), catenin beta 1 (CTNNB1), erb-b2 receptor tyrosine kinase 4 (ERBB4), F-box and WD repeat domain containing 7 (FBXW7), fms related receptor tyrosine kinase 3 (FLT3), serine/threonine kinase (LKB1), phosphatidylinositol-4,5-bisphosphate 3-kinase catalytic subunit alpha (PIK3CA).

### 2.2 Generation of lung cancer xenografts

PDXs were generated as previously described (24). Once mice developed tumor, they were sacrificed by cervical dislocation. The tumors were harvested by dissection and fixed in formalin and embedded in paraffin for histology and immunohistochemistry analyses. All procedures involving animals and their care conformed to institutional guidelines that comply with national and international laws and policies (EEC Council Directive 86/609, OJ L 358, 12 December 1987) and were authorized by the Italian Ministry of Health.

### 2.3 Histology and immunohistochemistry

Four-micron-thick formalin-fixed, paraffin-embedded (FFPE) tumor samples were stained either with hematoxylin and eosin or processed for IHC, which was performed by using the automatic stainer BOND III, (Leica Microsystems, Wetzlar, Germany). The following antibodies were used, according to the manufacturer’s instructions: anti-ACC Rabbit mAb detecting all isoforms of human ACC (clone C83B10, Cell Signaling Technology, dilution 1:100), anti-CPT1A Goat pAb (Novus Biologicals, dilution 1:300), anti-FAS Rabbit mAb (clone C20G5, Cell Signaling Technology, dilution 1:100), anti-GLS Rabbit mAb (clone EP7212, Abcam, dilution 1:200), anti-Ki67 (clone MIB-1, Dako Omnis, dilution 1:50), anti-LKB1 Mouse mAb (clone Ley 37D/G6, Santa Cruz Biotechnology, dilution 1:100), anti-MCT4 Mouse pAb (clone D-1, Santa Cruz Biotechnology, dilution 1:200) and anti-CD31 Rat mAb (clone SZ31, DIANOVA, dilution 1:40). Liquid diaminobenzidine (DAB; Bond Polymer Refine Detection, Leica Biosystems, Newcastle, UK) was used as a chromogenic agent and sections were counter-stained with Mayer’s hematoxylin. For the double staining with the anti-GLS Rabbit mAb and anti-MCT4 Rabbit pAb the DAB chromogenic agent and the Green chromogen (Bond Polymer Refine HRP-PLEX Detection, Leica Biosystems, Newcastle, UK) were used in a sequential assay (further details are listed in **Table 1, Supplementary Materials**).

### 2.4 Image acquisition and analysis

Tumor representation and quality of staining were initially evaluated by one experienced pathologist (GE). Slides were digitally acquired at 200x magnification by the Aperio CS2 (Leica Biosystems, Wetzlar, Germany) and the evaluation of IHC score was assessed through the ScanScope Image Analysis software (ImageScope v12.4.0.708). On the basis on their localization, the different markers were analyzed by using the Aperio membrane algorithm v9 (MCT4), the Aperio cytoplasmic algorithm v2 (GLS, CPT1A, FAS, ACC, LKB1), the Aperio nuclear algorithm (Ki67) and the microvessel analysis v1 (CD31), as previously described (18). Aperio Genie Classifier was trained to recognize tumor tissue, stroma and background (glass) and then combined with Aperio Membrane v9 and Aperio Cytoplasmic v9. Results provided the percentage of cells with different expression levels of proteins classified in 3+ (highly positive), 2+ (intermediate positive), 1+ (low positive) and 0 (negative) in the case of MCT4, ACC, LKB1 and Ki67 markers. The sum of percentage of marker positive cells for these 4 tiers equals 100%. Due to the lack of differences in intensity of expression, in the case of GLS, FAS and CPT1A markers results provide the percentage of marker positive cells (1+). Digital quantification performed by the software was verified by the pathologist (GE). Expression of the metabolism-associated markers was calculated according to the H-score system (reviewed in (25):), using as input digital pathology data, and values range between 0 and 300.

### 2.5 Statistical analysis

Data were analyzed with RStudio (RStudio: Integrated Development for R. RStudio Inc., Boston, MA, US). Quantitative variables were summarized as median and interquartile range. A descriptive analysis of the strength of relationship between the levels of all the considered markers was performed using the Spearman rank correlation coefficient. A two-tailed Mann-Whitney test was used to address the comparisons of each marker distribution between TP53, KRAS, LKB1 mutational status and between maximal growth inhibition levels (≤50% vs >50%). Survival times were estimated with the Kaplan Meier method and compared among groups of markers with the log-rank test. Each marker was dichotomized with cut-off corresponding to the most significant relation with the outcome, estimated from maximally selected log-rank statistics from the ‘maxstat’ R package and the association with the outcome was tested in univariate Cox proportional hazards regression models. P-values were not adjusted for multiple comparisons.

## 3 Results

### 3.1 Selection of metabolism-associated markers and panel set-up

We analyzed by IHC expression of the following markers in PDX sections: MCT4, GLS, CPT1A, FAS, and ACC. These markers identify key transporters or enzymes involved in glycolysis (MCT4), glutamine (GLS), and fatty acid metabolism (CPT1A, FAS, ACC). For all markers analyzed, the algorithm first identifies tumor cells and then quantifies expression levels according to the tiering system described in the methods section.

**Figure 1:**
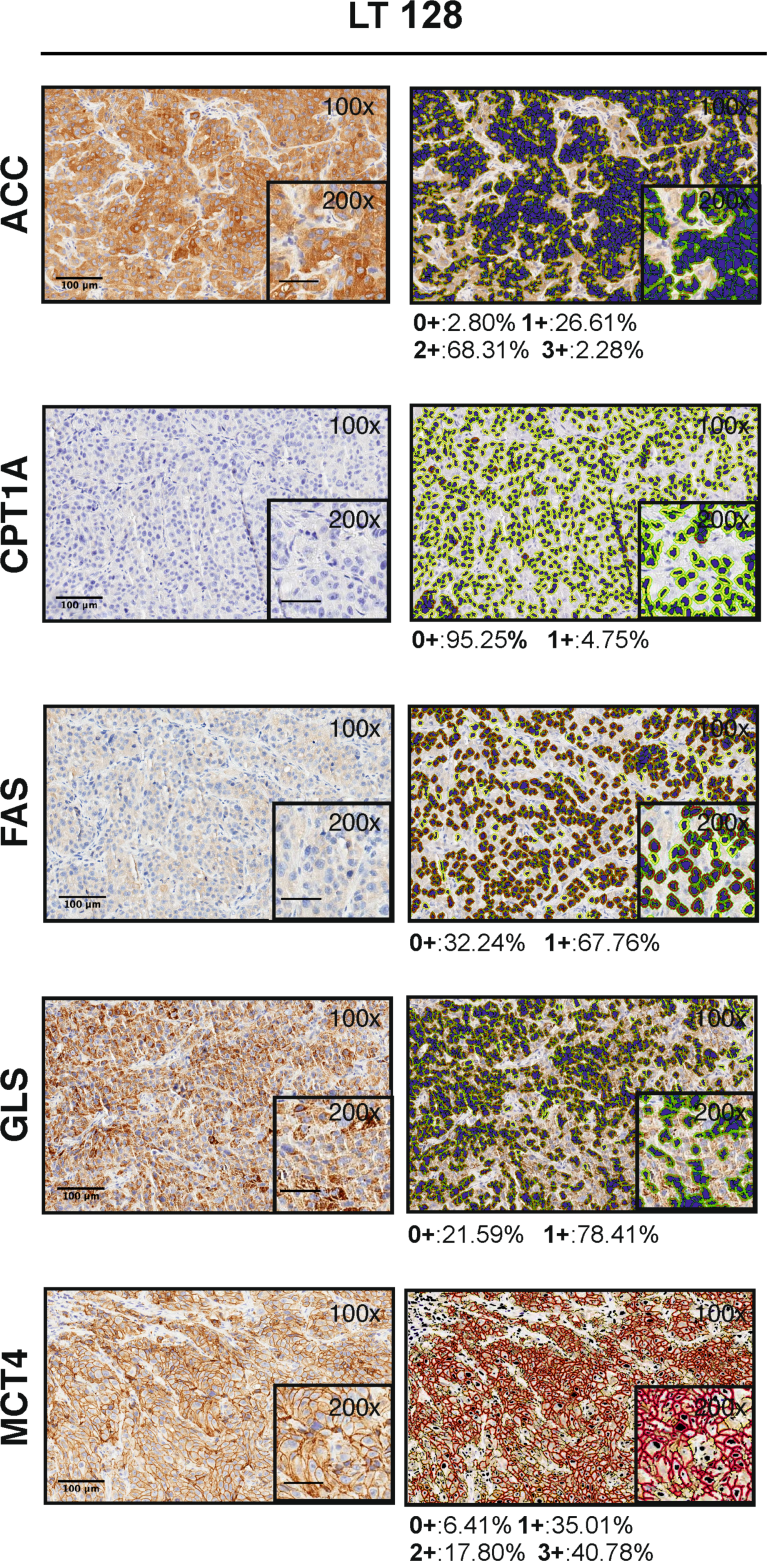
Visualization of the Aperio algorithms used to quantify metabolic markers expression in tissue (original magnification 100x and 200x, scale bar represents 100 μm or 50 μm, respectively). On the left, representative images of PDX LT 128 sections stained for ACC, FAS, GLS, MCT4 and CPT1A (hematoxylin counterstain). On the right, images showing the final mark-up of the sample. Quantification of the marker expression is shown below each panel: positive (1+/brown) or negative (0+/yellow) for CPT1A, FAS and GLS staining; strong (3+/brown or red), moderate (2+/orange), or weak (1+/yellow) for ACC and MCT4 staining. Negative/0+ cells show only hematoxylin counterstain.

**Figure 2:**
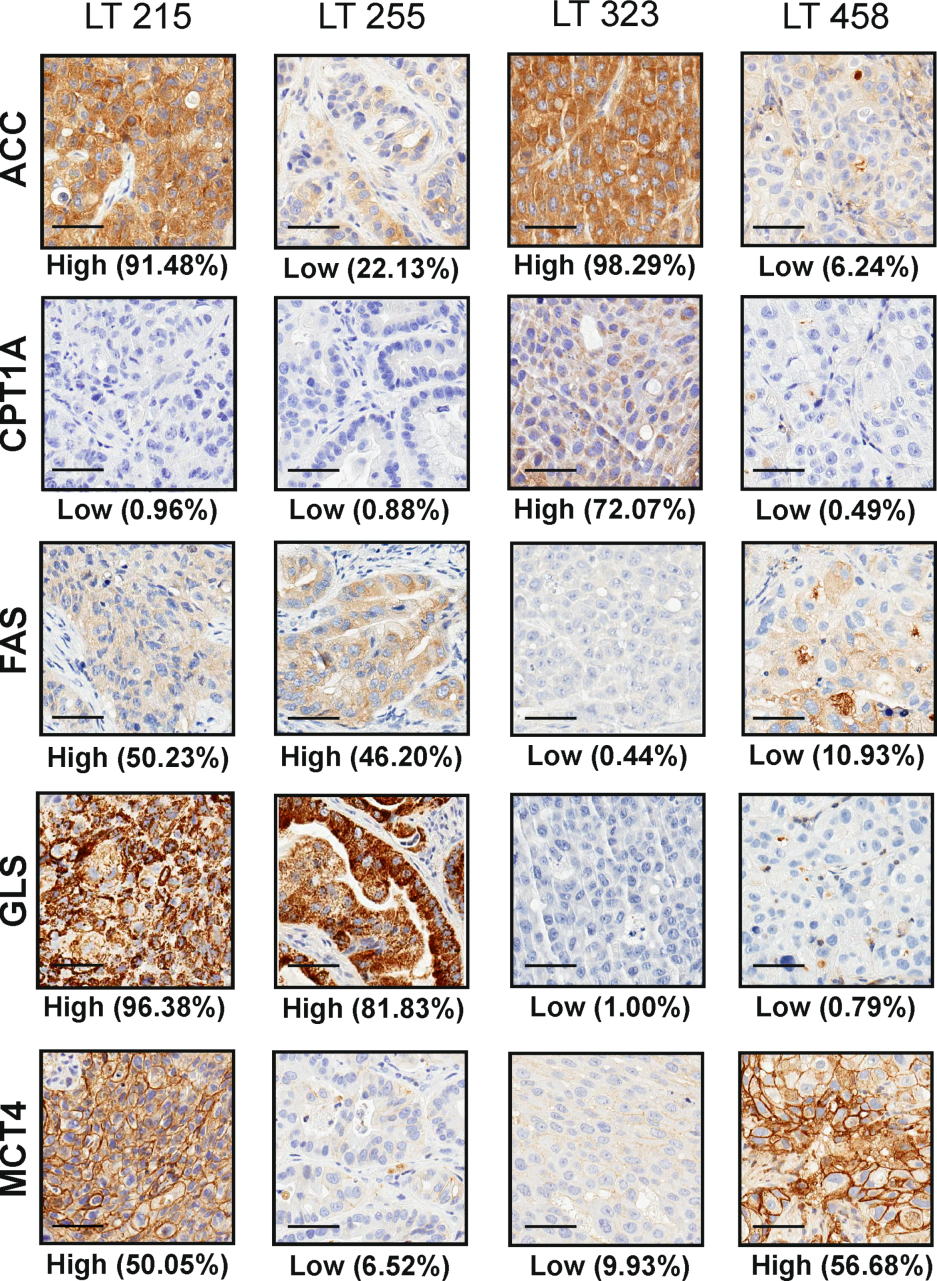
Representative pictures of four PDX (LT 215, LT 255, LT 323 and LT 458) stained for the selected metabolism-associated markers: ACC, CPT1A, FAS, GLS and MCT4 (original magnification 200x, scale bar represents 50 μm). The labels “High” and “Low” below each panel indicate the stratification of the sample according to the median value of expression of the marker according to digital pathology analysis, as detailed in the M&M section.

We performed IHC and digital pathology analysis of the expression of the five metabolism-associated markers in all 17 PDX samples (**Table 2; Supplementary materials**). Representative pictures of one PDX sample stained for each marker and the related mark-up of the analyzed tissue are presented in **Figure 1**. Detailed digital pathology results are presented in **Table 2**, whereas some representative pictures showing expression of the five markers in PDX samples are shown in **Figure 2**.

**Table 2:** Quantification of marker expression in lung adenocarcinoma PDXs by digital pathology.

A. Expression levels of metabolism-associated markers MCT4, GLS, ACC, FAS and CPT1A					
PDX ID	MCT4	GLS	ACC	FAS	CPT1A
LT 431	127.07	259.22	75.18	88.98	35.83
LT 323	55.35	3.01	253.65	1.33	216.22
LT 215	163.10	289.14	212.41	150.69	2.89
LT 141	106.36	155.91	18.02	4.98	2.35
LT 497	63.05	NA	2.41	0.91	0.77
LT 305	80.75	0.26	180.92	60.39	NA
LT 278	20.44	25.72	23.73	15.91	99.21
LT 265	153.25	147.56	222.45	39.22	25.49
LT 138	75.83	282.47	176.96	299.15	240.00
LT 273	109.68	197.16	187.33	0.25	9.62
LT 255	61.00	245.48	110.97	138.61	2.67
LT 267	160.74	289.78	222.25	8.68	32.23
LT 458	181.75	2.37	104.88	32.78	1.50
LT 66	181.04	245.39	290.98	297.84	142.99
LT 128	192.95	235.22	170.05	203.29	14.25
LT 111	101.63	236.32	46.69	31.36	6.98
LT 220	108.74	221.59	99.07	11.49	93.10
***Median***	**108.74**	**228.41**	**170.05**	**32.78**	**19.87**
B. Expression levels of CD31, LKB1 and the proliferation marker Ki67					
PDX ID	CD31	LKB1		Ki67	
LT 431	53.2	259.99		164.04	
LT 323	45.0	126.88		53.99	
LT 215	64.8	197.71		193.55	
LT 141	77.3	148.07		98.91	
LT 497	105	NA		72.15	
LT 305	26.3	96.01		80.34	
LT 278	270	230.48		121.42	
LT 265	67.5	179.81		119.03	
LT 138	280	NA		130.22	
LT 273	120	130.94		92.20	
LT 255	220	192.79		84.34	
LT 267	54.1	186.90		109.04	
LT 458	NA	114.58		68.30	
LT 66	200	181.59		122.25	
LT 128	75.8	108.79		138.14	
LT 111	82.8	182.17		92.61	
LT 220	132	295.27		173.71	
***Median***	**80.05**	**181.59**		**109.04**	

NA, Not Available. Values are expressed according to the H-score system. Bold, median values.

With regard to the pattern of expression of these metabolism-associated markers in tumors, we found intra-tumor heterogeneity in three out of seventeen PDX samples stained with MCT4 (LT 111, LT 255, LT 431) and one PDX sample stained with GLS (LT265). In all other cases, heterogeneous expression of the metabolic markers was not observed. Representative pictures illustrating heterogeneous expression of MCT4 and GLS are shown in **Supplementary Figures 1** and **2**. Notably, in 2 out of 3 PDX samples, MCT4-positive cells were located near the necrotic areas, likely underscoring the well-known hypoxia-driven upregulation of MCT4 expression. We conclude that intra-tumor heterogeneity occurs at a limited degree for most of the markers analyzed.

Considering the possible heterogeneous expression of these markers in different tumor samples, to assess the consistency of our findings, we performed IHC staining of MCT4, GLS, ACC, FAS and CPT1A in 8 additional samples obtained by implantation of the same specimen (LT66, LT111, LT141, LT 220, LT255, LT267, LT278, LT458) in different mice. Quantification of the markers and statistical analysis by the Wilcoxon test did not show significant differences between replicates for each biomarker, except for FAS (p-value 0.01).

Based on median values of the H-score, the metabolism-associated marker most abundantly expressed in these PDX samples was GLS (228.41), followed by ACC (170.05), MCT4 (108.74), FAS (32.78) and CPT1A (19.87). For descriptive purposes, we stratified PDX samples into two groups (high and low), based on the expression of each marker above or below the median value. We defined as plurimetabolic those PDX which showed high expression of the glycolysis-associated marker MCT4 in combination with one or more of the OXPHOS-associated markers (GLS, FAS, ACC, CPT1A). Plurimetabolic PDX (n = 9, 53%) included LT66, LT128, LT215, LT220, LT265, LT267, LT273, LT431 and LT458.

**Figure 3:**
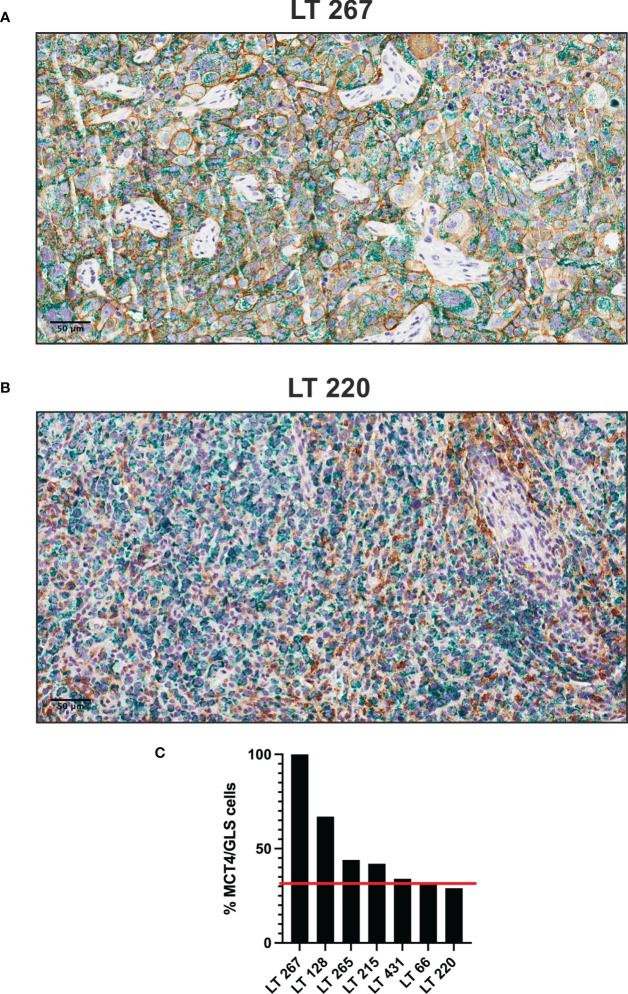
Plurimetabolic PDX showing expression of MCT4 (membrane, brown staining) and GLS (cytoplasm, green staining) by the tumor cells (two representative pictures in panel **(A, B)**, original magnification 200x, scale bar represents 50 μm). Relative percentage of PDX’s double stained (MCT4/GLS) cells is reported as histogram **(C)**. Red line indicates the median value of MCT4/GLS co-expression (32%).

To investigate the pattern of expression of some these markers in plurimetabolic tumors, we set-up double IHC for MCT4 and GLS. Interestingly, plurimetabolic PDX samples showed co-expression of the two markers by variable proportions of tumor cells, as shown in **Figure 3**. In some PDX (LT 267 and LT128) a substantial (>50%) of cells co-expressed MCT4 and GLS. In all other plurimetabolic PDX, however, MCT4 and GLS were expressed mainly by different tumor cells which were located in the same spatial region of the tumor (**Figure 3**).

Six out of 17 samples (35%) were classified OXPHOS, as they expressed OXPHOS markers but not MCT4 and included LT111, LT138, LT255, LT278, LT305 and LT323.

Altogether, these results suggest that OXPHOS is the main metabolic pathway sustaining energy and macromolecules production in the large majority (15/17, 88%) of these lung adenocarcinoma PDX samples, alone or in combination with glycolysis. Finally, two PDX (LT497 and LT141) expressed all markers at relatively low levels, suggesting utilization of other metabolic pathways not covered by the IHC panel.

### 3.2 Association between markers

As the markers selected identify key metabolic processes, it was interesting to investigate possible associations between the markers. This analysis disclosed that ACC was positively associated with CPT1A (r = 0.47) with borderline significance (**Table 3**), fitting the known biochemical role of these enzymes in FA metabolism (26). No other associations were found between the other metabolic markers analyzed (**Table 3**).

**Table 3:** Association between the analyzed markers.

Parameter1	Parameter2	rho	95% CI	p
MCT4	GLS	0.24	[-0.31, 0.66]	*0.3804*
MCT4	ACC	0.34	[-0.18, 0.71]	*0.1809*
MCT4	LKB1	-0.18	[-0.64, 0.38]	*0.5243*
MCT4	Ki67	0.39	[-0.13, 0.74]	*0.1220*
MCT4	FAS	0.34	[-0.19, 0.71]	*0.1842*
MCT4	CPT1A	-0.16	[-0.61, 0.38]	*0.5643*
MCT4	CD31	-0.26	[-0.68, 0.28]	*0.3218*
GLS	ACC	0.12	[-0.41, 0.59]	*0.6643*
GLS	LKB1	0.59	[0.09, 0.85]	***0.0208***
GLS	Ki67	0.59	[0.11, 0.84]	***0.0165***
GLS	FAS	0.41	[-0.12, 0.76]	*0.1102*
GLS	CPT1A	0.11	[-0.44, 0.60]	*0.7039*
GLS	CD31	0.17	[-0.38, 0.64]	*0.5327*
ACC	LKB1	-0.29	[-0.71, 0.28]	*0.2957*
ACC	Ki67	0.04	[-0.46, 0.52]	*0.8738*
ACC	FAS	0.27	[-0.26, 0.67]	*0.2999*
ACC	CPT1A	0.47	[-0.05, 0.79]	*0.0639*
ACC	CD31	-0.29	[-0.70, 0.25]	*0.2739*
LKB1	Ki67	0.62	[0.15, 0.87]	***0.0127***
LKB1	FAS	0.05	[-0.49, 0.56]	*0.8595*
LKB1	CPT1A	0.26	[-0.33, 0.70]	*0.3664*
LKB1	CD31	0.38	[-0.20, 0.77]	*0.1745*
Ki67	FAS	0.52	[0.04, 0.81]	***0.0325***
Ki67	CPT1A	0.38	[-0.16, 0.74]	*0.1472*
Ki67	CD31	0.16	[-0.38, 0.62]	*0.5569*
FAS	CPT1A	0.27	[-0.28, 0.68]	*0.3163*
FAS	CD31	0.18	[-0.36, 0.63]	*0.5061*
CPT1A	CD31	0.11	[-0.44, 0.60]	*0.6945*

Association between the different analyzed markers using the Spearman correlation test. p-value adjustment method: none. Bold, statistically significant values (p<0.05).

CPT1A levels were significantly higher in KRAS-wt NSCLC PDX samples (p=0.010). No further associations between the metabolism-associated markers and recurrent TP53 and LKB1 gene mutations were found.

Finally, since alterations of LKB1 are relatively common in lung adenocarcinoma (27), and in view of the established role of this serine/threonine kinase in the regulation of metabolism (28), we assessed LKB1 expression in these tumors by IHC (**Supplementary Figure 3**). Among PDX samples with low LKB1 expression, three (LT 128, LT273 and LT 458) were known to bear disruptive LKB1 mutations (**Table 1**), whereas two (LT 305 and LT323) had LKB1 WT sequence, suggesting epigenetic down-regulation of LKB1 protein expression. Interestingly, we found a significant positive association between LKB1 and GLS expression (r= 0.59, **Table 3**).

### 3.3 Association with proliferation and angiogenesis

Next, we investigated whether expression of any of these markers was associated with proliferation, in view of the well-established link between certain metabolic processes and proliferation (29). We found that the proliferation marker Ki67 in tumor sections was positively associated with GLS (r=0.59) and FAS (r=0.52). In contrast, no association was found between Ki67 and any of the other metabolic markers; LKB1 was also positively associated with Ki67 in this dataset (r = 0.62) (**Table 3**).

Angiogenesis is a biological process which strongly contributes to tumor growth and is partially regulated by metabolic features of tumors, such as the production of lactate by highly glycolytic tumors (30). We analyzed possible associations between expression levels of the five metabolism-associated markers and microvessel density (MVD), calculated based on quantification of CD31 positive cells, as readout of angiogenesis (**Supplementary Figure 3**). Results, however, did not disclose any significant association (**Table 3**), perhaps due to the low number of samples analyzed.

### 3.4 Association with the kinetics of tumor growth

**Figure 4:**
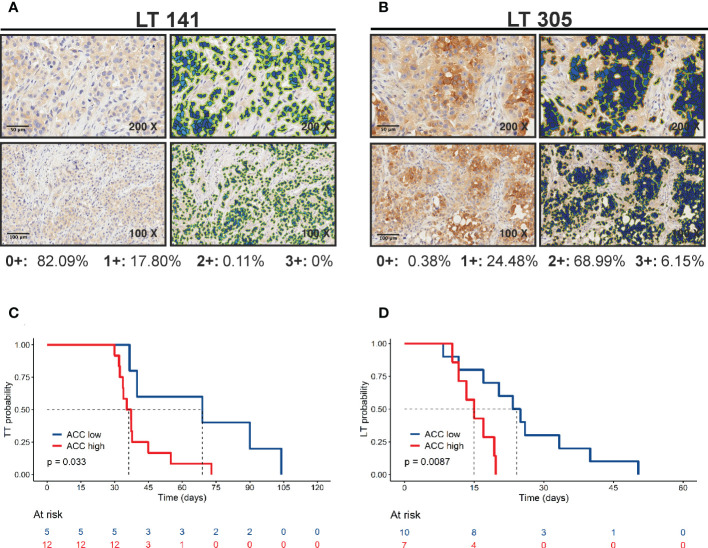
Positive correlation of ACC expression with tumor growth. **(A, B)** ACC expression was quantified by Aperio Cytoplasmic v9 algorithm. The panels show representative samples with low (LT 141) and high (LT 305) ACC expression levels (original magnification 200x for upper panels or 100x for lower panels, scale bar represents 50 or 100 μm, respectively). **(C)** Kaplan-Meier curves show faster growth of PDXs with high ACC expression levels. The best cut-off value used to generate this curve was 75.176. Statistical significance was calculated with the log-rank test **(D)** Kaplan-Meier curves show shorter latency time of PDXs with high ACC expression levels. The best cut-off value used to generate this curve was 176.96. Statistical significance was calculated with the log-rank test.

Finally, we investigated the association between the metabolism-associated markers and the kinetics of tumor growth in mice, indicated by the parameter time to transplantation (TT – time from implant to explant) and the initial latency time (LT) (**Table 4A, B**, respectively). For this analysis, PDX samples were stratified into two groups based on the value of the marker obtained by maximizing its discriminative ability (best cut-off). The only marker positively associated with tumor growth in mice was ACC (**Table 4** and **Figure 4**). Among other markers analyzed, LKB1 was not associated with TT.

**Table 4:** Association of the metabolic-associated markers with the kinetics of tumor growth.

A. Association with TT (time from implant to explant)									
	IHC-Expression Cut-off		N	Median TT (95%CI)	logrank		HR (95%CI)		p-value
MCT4	106.358	Above cut-off	9	37.3 (30.0;55)	*0.3237*		Ref		
		Below cut-off	8	38.9 (32.3;73)			0.62 [0.23;1.63]		*0.3265*
GLS	245.479	Above cut-off	4	55.2 (37.3;NA)	*0.3505*		Ref		
		Below cut-off	12	36.0 (32.0;55)			1.62 [0.56;5.55]		*0.3871*
ACC	75.176	Above cut-off	12	36.4 (32.0;45)	***0.0327***		Ref		
		Below cut-off	5	69.0 (36.7;NA)			0.29 [0.07;0.91]		***0.0330***
FAS	1.325	Above cut-off	14	37.5 (32.3;69)	*0.2968*		Ref		
		Below cut-off	3	35.4 (33.9;NA)			2.21 [0.54;7.30]		*0.2464*
CPT1A	2.354	Above cut-off	13	36.7 (32.3;55)	*0.1641*		Ref		
		Below cut-off	3	45.0 (40;NA)			0.43 [0.08;1.45]		*0.1878*
CD31	0.00022	Above cut-off	2	71 (69.0;NA)	*0.3590*		Ref		
		Below cut-off	14	37 (32.3;40)			1.70 (0.48;8.94)		*0.4365*
B. Association with LT (latency time)									
	IHC-Expression Cut-off	Below cut-off	N	Median TT (95%CI)	logrank		HR (95%CI)		p-value
MCT4	101.627	Above cut-off	10	20 (10.3; 26.0)		*0.2903*		Ref		
		Below cut-off	7	17 (8.3; 23.3)			1.73 [0.62; 4.72]		*0.2849*
GLS	245.479	Above cut-off	4	26.5 (11.7;NA)		*0.4658*		Ref		
		Below cut-off	12	18.2 (10.3;25)				1.43 [0.49;4.96]		*0.5209*
ACC	176.960	Above cut-off	7	15.0 (10.3;19.3)		***0.0009***		Ref		
		Below cut-off	10	24.2 (8.3;33.3)				0.21 [0.05;0.72]		***0.0135***
FAS	1.326	Above cut-off	14	20 (11.7;26)		*0.2276*		Ref		
		Below cut-off	3	17 (13.3;NA)				2.44 [0.58;8.66]		*0.2061*
CPT1A	2.354	Above cut-off	13	19.3 (11.7;23.3)		*0.1957*		Ref		
		Below cut-off	3	25.0 (17.0;NA)				0.46 [0.09;1.56]		*0.2350*
CD31	0.000077	Above cut-off	8	17.0 (8.33;23.3)		*0.3226*		Ref		
		Below cut-off	8	19.5 (10.33;40.0)			0.58 (0.20;1.70)		*0.3200*

The TT parameter (time from implant to explant) was used for association analysis. HR, Hazard Ratio; N, number of mice.

The LT parameter (latency time) was used for association analysis. HR, Hazard Ratio; N, number of mice. Bold, statistically significant p and logrank values.

Finally, we tested the correlation between marker expression and tumor volume at sacrifice calculating Spearman’s rank correlation coefficient. We included in the analysis all samples available (n=24, including also the PDX specimens with two replicates). MCT4, ACC, FAS and CPT1A have a weak to moderate correlation (r MCT4 = 0.26, r GLS = 0.20, r ACC = 0.43, r FAS = 0.35, r CPT1A = 0.26) and ACC was the only marker statistically significant (p = 0.03), in line with the above results of correlations with LT and TT parameters. Moreover, plurimetabolic PDX positively correlated with tumor volume at sacrifice: the median tumor size for pluri-metabolic PDX was 700 mm^3^ (n = 11), versus a median tumor size of 500 mm^3^ (n= 13) for the OXPHOS group (p = 0.03, Mann-Whitney test).

## 4 Discussion

PDX are a valuable resource both to understand the biological complexity of cancer and to investigate new therapeutic approaches in pre-clinical models considered closer to the patients compared with other available *in vivo* models. PDXs have been reported for many types of solid tumors (31). In the case of lung cancer, several groups established PDX models (32–36) and reported their genetic fingerprint (23, 37). This associated genetic information was key to the discovery of new targets, such as the identification of HER2 as an effective therapeutic target in cetuximab-resistant colorectal cancer (38). In all previous studies, however, there is a lack of information about the metabolic features of PDXs. This gap may limit testing of metabolism targeting drugs, whose therapeutic activity is likely dependent on the metabolic set-up of tumor cells, as shown by several recent studies (39). Moreover, it appears that specific metabolic activities of cancer cells also modulate response to conventional chemotherapies (8) and therapeutic resistance (39, 40), suggesting that metabolic features should be taken into consideration when new drugs are tested in PDX models.

Although comprehensive metabolic portraits of tumors are best obtained through mass-spectrometry-based analysis, we propose a simplified metabolic classification of tumors using quantitative measurement of the expression at protein level of well-established enzymes or transporters involved in key metabolic pathways. In this pilot study, we tested the feasibility of this approach in a small subset of NSCLC PDX, previously established and characterized by M. Moro et al. (23). These NSCLC PDX retained the key genetic alterations of the matched patients tumor samples and were also shown to reproduce their main metabolic features, as shown by [18F]FDG PET imaging studies (41).

Our results indicate that the majority of the analyzed models rely on OXPHOS metabolism, either alone or in combination with glucose metabolism. Notably, 9 out of 17 PDX showed a plurimetabolic phenotype, with co-expression of MCT4 and GLS at high (above the median value) levels. Double IHC disclosed that in some plurimetabolic PDX the two markers were co-expressed by the same tumor cells, whereas in others MCT4 and GLS were mostly expressed by different cells which, however, clustered in the same spatial area of the tissue. While plurimetabolic tumors have been identified also by using other techniques, such as single cell RNAseq (9), imaging of metabolism-associated markers by quantitative IHC has the advantage to enable spatial localization of the two signals. This feature will likely be increasingly important in future studies to better understand metabolic heterogeneity of tumors. We highlighted a correlation between LKB1, GLS and Ki67 expression levels, although we did not explore the exact spatial localization of Ki67+ cells and that of the other associated markers, which represents a limitation of our pilot study. LKB1 is a tumor suppressor that acts by suppressing growth under energetic stress conditions, through its action on the AMPK/mTOR pathway (28). A positive correlation between LKB1 and Ki67 may seem counter-intuitive, especially for tumors that grow under the pressure of a non-orthotopic murine microenvironment. Nevertheless, our observation suggests that LKB1 wild type NSCLC rely on glutamine consumption to sustain the proliferation induced by hyperactivation of oncogenes such as KRAS (**Table 1**). Glutamine sustains cell growth as a nitrogen source for the synthesis of purine and pyrimidine bases, and it is also important for maintaining redox homeostasis within highly proliferating cells (42). Furthermore, a strong correlation of glutamine dependency with cell proliferation has been observed in a landmark study involving hundreds of cancer cell lines (43). A higher dependency on glutamine-related ROS detoxyfication activity has been reported for KRAS/LKB1/KEAP1 triple mutants compared to KRAS/LKB1- or KRAS-mutated NSCLC (44). However, in the same manuscript a reduction of ATP production upon glutaminase inhibition was reported also in LKB1 wild type NSCLC cells. ATP reduction leads to cell growth arrest through activation of the LKB1/AMPK pathway. Since the energetic stress induced by biguanides in LKB1 mutated NSCLC models has been reported to cause apoptotic cell death (45), inhibiting at the same time GLS and AMPK activity may represent a new therapeutic option to investigate in NSCLC LKB1 wild type preclinical models.

Of note, we reported here a correlation between PDX *in vivo* growth rate, measured as time-to-transplantation (TT) and ACC expression. PDXs grow *in vivo* with a two-step kinetic; after an initial latency time (LT) tumors start growing “exponentially”. LT may be considered as a measure of the time needed by tumor cells to productively interact with the murine microenvironment. Both LT and exponential growth contribute to TT. Interestingly, correlation between LT and ACC expression was also significant (**Figure 4**). Thus, ACC seems to be related to tumor growth when these interactions are being established. Speculatively, lipid metabolism within PDXs may be important for *in vivo* increase in tumor growth by inducing the recruitment of stromal cells, such as fibroblasts and innate immune cells. Alternatively, ACC could modulate cell autonomous features of cancer cells, although ACC expression did not correlate with cell proliferation markers (Ki67). Finally, lipid metabolism could regulate tumor cell apoptosis, which we did not evaluate in these samples. As note of cautiousness, given the small number of PDX analyzed, hypothesis about the prognostic role of ACC in NSCLC need to be confirmed by larger studies involving either PDX or patients’ samples.

In conclusion, results of this pilot study support the feasibility of a metabolic classification of NSCLC PDXs based on quantification of IHC markers by digital pathology. There are, however, some intrinsic limitations of our study, including the small number of PDX samples and markers analyzed, the limited investigation of the spatial distribution of the different markers and the lack of orthogonal validation of findings by state-of-the-art techniques used to study tumor metabolism. Additional constraints are represented by the lack of comparison between the PDX and the original primary tumor and lack of validation between the metabolic characterization and response to metabolic therapies. Therefore, additional studies will be required to consolidate these preliminary results and identify additional markers for a refinement of the proposed panel. Finally, larger cohorts of samples need to be analyzed to establish the possible prognostic or predictive value of these metabolism-associated markers in NSCLC.

## Data availability statement

The original contributions presented in the study are included in the article/**Supplementary Material**. Further inquiries can be directed to the corresponding author.

## Ethics statement

Approval of the research protocol by an Institutional Reviewer Board: The study was conducted in accordance with the Declaration of Helsinki, and approved by the Institutional Review and the Ethics Boards of the Fondazione IRCCS Istituto Nazionale Tumori.

## Author contributions

Conceptualization, EZ and SI. Methodology, FF, EZ, MM, TZ, and GE. Software, FF and PD. Validation, FF. Formal analysis, FF and PD. Resources, SI. Data curation, SI. Writing—original draft preparation, SI. Writing—review and editing, SI, MM, CB, GS, EZ, and FF. Visualization, SI. Supervision, SI. Project administration, SI. Funding acquisition, SI. All authors contributed to the article and approved the submitted version.
