# Supplementary material for: Metabolic classification of non-small cell lung cancer patient-derived xenografts by a digital pathology approach: A pilot study
Source: Front Oncol. 2023 Feb 28;13:1070505. doi: 10.3389/fonc.2023.1070505 (PMC10011479; doi:10.3389/fonc.2023.1070505)
Supplement: Supplementary file 1 [file DataSheet_1.docx]

Supplementary Material

**Supplementary Table 1.** IHC reagents and protocols.

| **Epitope** | **Heat-Induced**  **Epitope Retrieval** | **Primary antibody** | **Secondary antibody** | **HRP polymer** | **Detection** |
| --- | --- | --- | --- | --- | --- |
| ACC | 20’ at 100 °C with Citrate Based Solution, pH 6 (Epitope retrieval solution 1, AR9961, Leica) | clone C83B10, Cell Signaling Technology, dilution 1:100, 30’ at Room Temperature (RT), 3 Wash Stepss with Wash Solution (AR9590, Leica) | Post-primary (BOND Polymer Refine Detection System DS9800, Leica Biosystems) 8’ at RT, 3 Wash Stepss with Wash Solution (AR9590, Leica) | DAB Refine Substrate 10’, 3 wash stepss with water | Hematoxylin 8’, 3 wash stepss with water |
| CD31 Rat | 20’ at 100 °C with Citrate Based Solution, pH 6 (Epitope retrieval solution 1, AR9961, Leica) | clone SZ31, DIANOVA, dilution 1:40, 15’ at Room Temperature (RT), 3 Wash Steps with Wash Solution (AR9590, Leica) | Linker rabbit anti-rat (BA-4001, Vector) dilution 1:100, 15’, 3 wash stepss;  Post-primary (BOND Polymer Refine Detection System DS9800, Leica Biosystems) 8’ at RT, 3 Wash Steps with Wash Solution (AR9590, Leica) | DAB Refine Substrate 10’, 3 wash steps with water | Hematoxylin 5’, 3 wash steps with water |
| CPT1A | 10’ at 100 °C with Citrate Based Solution, pH 6 (Epitope retrieval solution 1, AR9961, Leica) | Novus Biologicals, dilution 1:300, 30’ at Room Temperature (RT), 3 Wash Steps with Wash Solution (AR9590, Leica) | Linker horse anti-goat (BA-9500, Vector), dilution 1:200, 15’, 3 wash stepss;  Post-primary (BOND Polymer Refine Detection System DS9800, Leica Biosystems) 8’ at RT, 3 Wash Steps with Wash Solution (AR9590, Leica) | DAB Refine Substrate 10’, 3 wash steps with water | Hematoxylin 10’, 3 wash steps with water |
| FAS | 20’ at 100 °C with Citrate Based Solution, pH 6 (Epitope retrieval solution 1, AR9961, Leica) | clone C20G5, Cell Signaling Technology, dilution 1:100, 15’ at Room Temperature (RT), 3 Wash Steps with Wash Solution (AR9590, Leica) | Post-primary (BOND Polymer Refine Detection System DS9800, Leica Biosystems) 8’ at RT, 3 Wash Steps with Wash Solution (AR9590, Leica) | DAB Refine Substrate 10’, 3 wash steps with water | Hematoxylin 10’, 3 wash steps with water |
| GLS | 10’ at 100 °C with Citrate Based Solution, pH 6 (Epitope retrieval solution 1, AR9961, Leica) | clone EP7212, Abcam, dilution 1:200, 15’ at Room Temperature (RT), 3 Wash Steps with Wash Solution (AR9590, Leica) | Post-primary (BOND Polymer Refine Detection System DS9800, Leica Biosystems) 8’ at RT, 3 Wash Steps with Wash Solution (AR9590, Leica) | DAB Refine Substrate 10’, 3 wash steps with water | Hematoxylin 10’, 3 wash steps with water |
| LKB1 | 30’ at 100 °C with Citrate Based Solution, pH 6 (Epitope retrieval solution 1, AR9961, Leica) | clone Ley 37D/G6, Santa Cruz Biotechnology, dilution 1:100, 30’ at Room Temperature (RT), 3 Wash Stepss with Wash Solution (AR9590, Leica) | Post-primary (BOND Polymer Refine Detection System DS9800, Leica Biosystems) 8’ at RT, 3 Wash Stepss with Wash Solution (AR9590, Leica) | DAB Refine Substrate 10’, 3 wash stepss with water | Hematoxylin 8’, 3 wash stepss with water |
| MCT4 | 20’ at 100 °C with Citrate Based Solution, pH 6 (Epitope retrieval solution 1, AR9961, Leica) | clone D-1, Santa Cruz Biotechnology, dilution 1:200, 15’ at Room Temperature (RT), 3 Wash Steps with Wash Solution (AR9590, Leica) | Post-primary (BOND Polymer Refine Detection System DS9800, Leica Biosystems) 8’ at RT, 3 Wash Steps with Wash Solution (AR9590, Leica) | DAB Refine Substrate 10’, 3 wash steps with water | Hematoxylin 10’, 3 wash steps with water |
| Double staining: I step_MCT4 | 20’ at 100 °C with Citrate Based Solution, pH 6 (Epitope retrieval solution 1, AR9961, Leica) | clone D-1, Santa Cruz Biotechnology, dilution 1:200, 15’ at Room Temperature (RT), 3 Wash Steps with Wash Solution (AR9590, Leica) | Post-primary (BOND Polymer Refine Detection System DS9800, Leica Biosystems) 8’ at RT, 3 Wash Steps with Wash Solution (AR9590, Leica) | DAB Refine Substrate 10’, 3 wash steps with water |  |
| Double staining: II step_GLS | 10’ at 100 °C with Citrate Based Solution, pH 6 (Epitope retrieval solution 1, AR9961, Leica) | clone EP7212, Abcam, dilution 1:200, 15’ at Room Temperature (RT), 3 Wash Steps with Wash Solution (AR9590, Leica) | Post-primary (BOND Polymer Refine Detection System DS9800, Leica Biosystems) 8’ at RT, 3 Wash Steps with Wash Solution (AR9590, Leica) | Green Chromogen (BOND Polymer Refine HRP PLEX Detection, DS9914) 8’, 3 wash steps with water | Hematoxylin 5’, 3 wash steps with water |

*****Ki-67 was performed by Ventana Benchmark Platform

**Supplementary Table 2.** Quantification of marker expression in lung adenocarcinoma PDXs by digital pathology.

|  | **MCT4** | | | | **GLS** | | **ACC** | | | | **FAS** | | **CPT1A** | |
| --- | --- | --- | --- | --- | --- | --- | --- | --- | --- | --- | --- | --- | --- | --- |
| **PDX ID** | **3+** | **2+** | **1+** | **0+** | **1+** | **0+** | **3+** | **2+** | **1+** | **0+** | **1+** | **0+** | **1+** | **0+** |
| LT 431 | 3.70 | 27.32 | 61.32 | 7.65 | 86.41 | 13.59 | 0.00 | 0.40 | 74.36 | 25.23 | 29.66 | 70.34 | 11.94 | 88.06 |
| LT 323 | 7.97 | 1.96 | 27.52 | 62.55 | 1.00 | 99.00 | 55.69 | 42.59 | 1.39 | 0.32 | 0.44 | 99.56 | 72.07 | 27.93 |
| LT 215 | 21.98 | 28.07 | 41.02 | 8.93 | 96.38 | 3.62 | 21.13 | 70.35 | 8.31 | 0.20 | 50.23 | 49.77 | 0.96 | 99.04 |
| LT 141 | 4.80 | 19.32 | 53.33 | 22.55 | 51.97 | 48.03 | 0.00 | 0.11 | 17.79 | 82.09 | 1.66 | 98.34 | 0.78 | 99.22 |
| LT 497 | 1.80 | 8.93 | 39.79 | 49.48 | NA | NA | 0.49 | 0.20 | 0.54 | 98.77 | 0.30 | 99.70 | 0.26 | 99.74 |
| LT 305 | 0.13 | 1.88 | 76.60 | 21.39 | 0.09 | 99.91 | 6.15 | 68.99 | 24.48 | 0.38 | 20.13 | 79.87 | NA | NA |
| LT 278 | 0.79 | 1.12 | 15.81 | 82.27 | 8.57 | 91.43 | 0.50 | 3.94 | 14.35 | 81.21 | 5.30 | 94.70 | 33.07 | 66.93 |
| LT 265 | 11.54 | 37.94 | 42.75 | 7.78 | 49.19 | 50.81 | 28.66 | 65.40 | 5.68 | 0.30 | 13.07 | 86.93 | 8.50 | 91.50 |
| LT 138 | 1.28 | 11.21 | 49.58 | 37.93 | 94.16 | 5.84 | 1.51 | 75.36 | 21.73 | 1.41 | 99.72 | 0.28 | 80.00 | 20.00 |
| LT 273 | 1.30 | 16.01 | 73.77 | 8.92 | 65.72 | 34.28 | 7.57 | 72.37 | 19.87 | 0.19 | 0.08 | 99.92 | 3.21 | 96.79 |
| LT 255 | 0.60 | 5.92 | 47.37 | 46.11 | 81.83 | 18.17 | 1.97 | 20.16 | 64.74 | 13.13 | 46.20 | 53.80 | 0.89 | 99.11 |
| LT 267 | 19.15 | 30.89 | 41.51 | 8.45 | 96.59 | 3.41 | 26.27 | 70.08 | 3.27 | 0.38 | 2.89 | 97.11 | 10.74 | 89.26 |
| LT 458 | 29.29 | 27.39 | 39.10 | 4.23 | 0.79 | 99.21 | 0.14 | 6.10 | 92.27 | 1.49 | 10.93 | 89.07 | 0.50 | 99.50 |
| LT 66 | 28.81 | 28.63 | 37.35 | 5.20 | 81.80 | 18.20 | 91.23 | 8.56 | 0.17 | 0.04 | 99.28 | 0.72 | 47.66 | 52.34 |
| LT 128 | 40.78 | 17.80 | 35.01 | 6.41 | 78.41 | 21.59 | 2.28 | 68.31 | 26.61 | 2.80 | 67.76 | 32.24 | 4.75 | 95.25 |
| LT 111 | 3.05 | 18.41 | 55.66 | 22.87 | 78.77 | 21.23 | 0.11 | 3.70 | 38.95 | 57.23 | 10.45 | 89.55 | 2.33 | 97.67 |
| LT 220 | 7.20 | 15.96 | 55.22 | 21.63 | 73.86 | 26.14 | 5.52 | 15.14 | 52.24 | 27.10 | 3.83 | 96.17 | 31.03 | 68.97 |
|  |  |  |  |  |  |  |  |  |  |  |  |  |  |  |

1. Expression level of metabolism-associated markers MCT4, GLS, ACC, FAS and CPT1A.
2. Expression Level of LKB1 and Ki67.

|  | **LKB1** | | | | | | | | | **Ki67** | | | | | |  |
| --- | --- | --- | --- | --- | --- | --- | --- | --- | --- | --- | --- | --- | --- | --- | --- | --- |
| **PDX ID** | | | **3+** | **2+** | | **1+** | | **0+** | | **3+** | | **2+** | | **1+** | **0+** | |
| LT 431 | | | 62.54 | 34.92 | | 2.53 | | 0.01 | | 38.32 | | 10.41 | | 28.28 | 23.00 | |
| LT 323 | | | 0.01 | 26.86 | | 73.13 | | 0.00 | | 3.79 | | 4.09 | | 34.44 | 57.68 | |
| LT 215 | | | 8.28 | 81.22 | | 10.44 | | 0.06 | | 49.90 | | 10.88 | | 22.08 | 17.14 | |
| LT 141 | | | 1.66 | 44.96 | | 53.18 | | 0.21 | | 9.47 | | 10.32 | | 49.85 | 30.36 | |
| LT 497 | | | NA | NA | | NA | | NA | | 17.13 | | 4.76 | | 11.24 | 66.87 | |
| LT 305 | | | 0.00 | 0.10 | | 95.80 | | 4.10 | | 6.53 | | 9.02 | | 42.72 | 41.73 | |
| LT 278 | | | 45.75 | 39.01 | | 15.21 | | 0.03 | | 13.23 | | 16.65 | | 48.43 | 21.69 | |
| LT 265 | | | 1.90 | 76.01 | | 22.09 | | 0.00 | | 19.32 | | 10.90 | | 39.29 | 30.50 | |
| LT 138 | | | NA | NA | | NA | | NA | | 21.46 | | 13.30 | | 39.27 | 25.98 | |
| LT 273 | | | 0.43 | 30.28 | | 69.09 | | 0.20 | | 15.04 | | 8.77 | | 29.53 | 46.66 | |
| LT 255 | | | 8.01 | 76.76 | | 15.22 | | 0.00 | | 8.07 | | 9.27 | | 41.60 | 41.07 | |
| LT 267 | | | 3.60 | 79.70 | | 16.69 | | 0.01 | | 14.55 | | 12.91 | | 39.56 | 32.98 | |
| LT 458 | | | 0.99 | 12.60 | | 86.41 | | 0.00 | | 9.37 | | 5.71 | | 28.75 | 56.16 | |
| LT 66 | | | 1.04 | 79.52 | | 19.44 | | 0.00 | | 22.51 | | 10.83 | | 33.07 | 33.59 | |
| LT 128 | | | 0.06 | 8.68 | | 91.25 | | 0.01 | | 27.08 | | 11.21 | | 34.49 | 27.22 | |
| LT 111 | | | 7.57 | 67.07 | | 25.30 | | 0.05 | | 10.15 | | 10.14 | | 41.86 | 37.85 | |
| LT 220 | | | 95.28 | 4.72 | | 0.01 | | 0.00 | | 38.65 | | 13.65 | | 30.44 | 17.25 | |
|  |  |  | | |  | |  | |  | |  | |  |  |  |  |
|  |  |  | | |  | |  | |  | |  | |  |  |  |  |
