# Supplementary figures and images for: Metabolic classification of non-small cell lung cancer patient-derived xenografts by a digital pathology approach: A pilot study
Source: Front Oncol. 2023 Feb 28;13:1070505. doi: 10.3389/fonc.2023.1070505 (PMC10011479; doi:10.3389/fonc.2023.1070505)

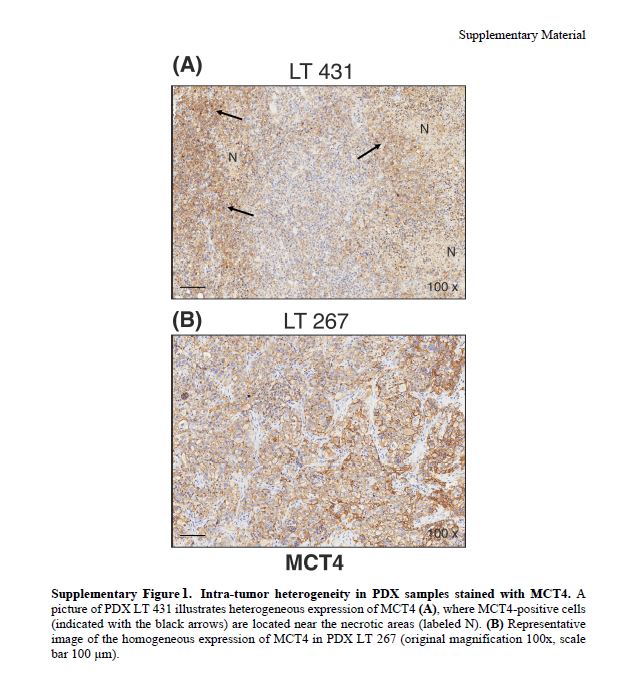

Supplement: Supplementary file 2 [file Image_1.jpg]

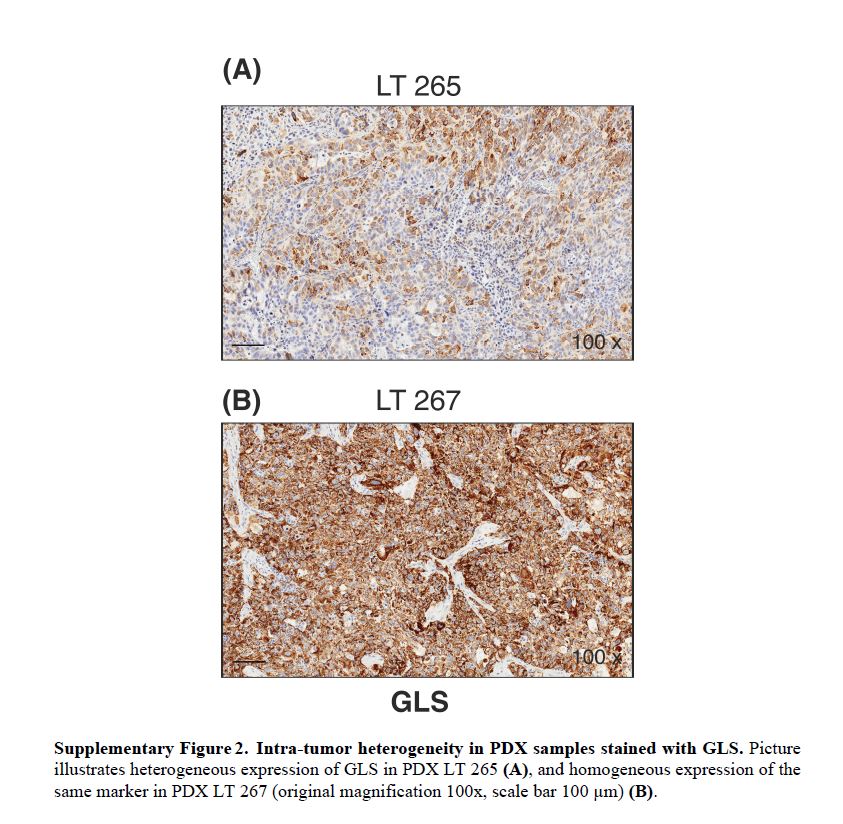

Supplement: Supplementary file 3 [file Image_2.jpg]
