# Supplementary material for: Metabolic classification of non-small cell lung cancer patient-derived xenografts by a digital pathology approach: A pilot study
Source: Front Oncol. 2023 Feb 28;13:1070505. doi: 10.3389/fonc.2023.1070505 (PMC10011479; doi:10.3389/fonc.2023.1070505)
Supplement: Supplementary file 4 [file Image_3.pdf]

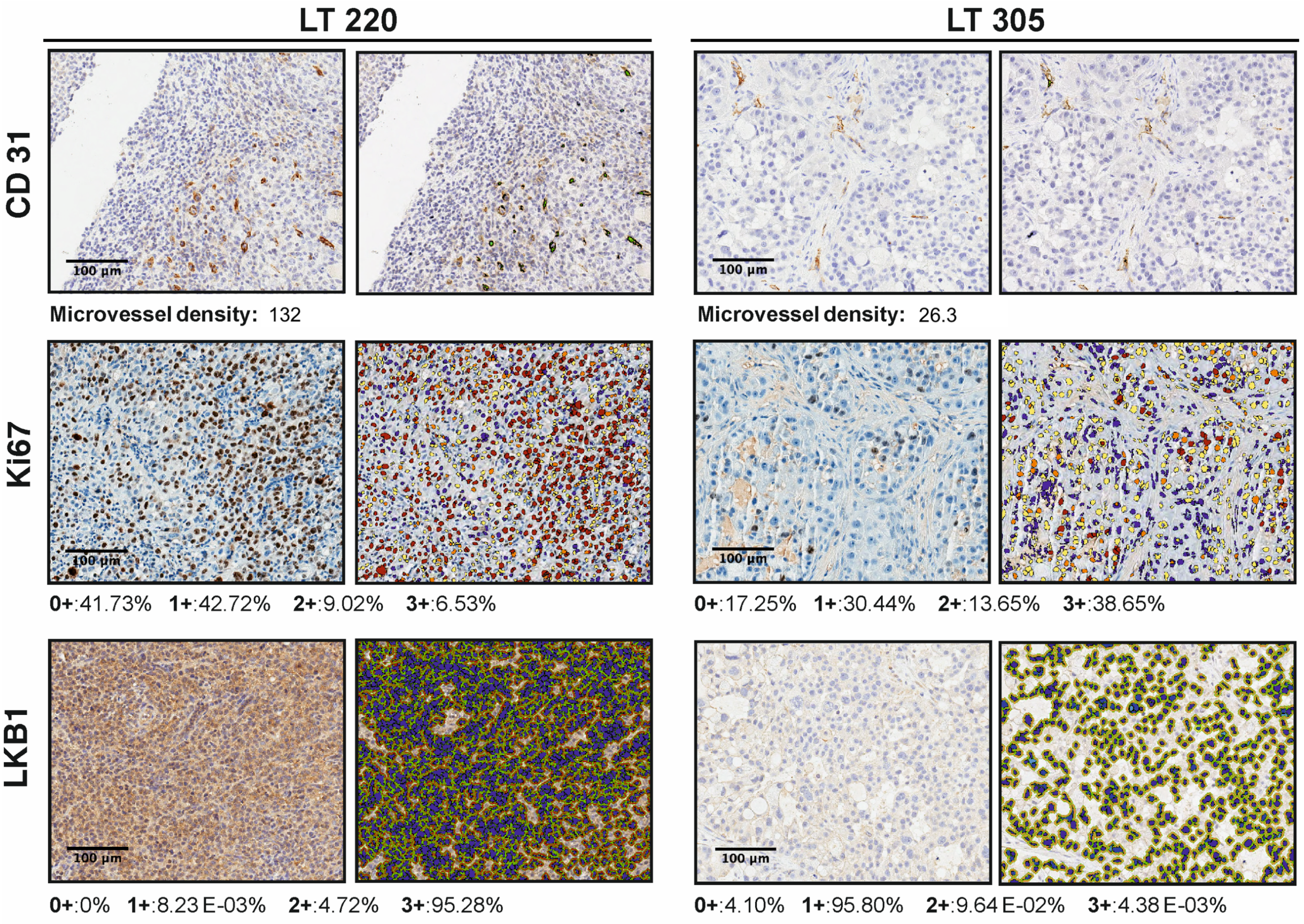

Supplementary Figure 3. Representative pictures of two PDX (LT 220 and LT 305) stained with markers CD31, Ki67 and LKB1 (original magnification 100x, scale bar 100 µm). The numbers below each panel indicate the percentage of strong (3+), moderate (2+) or weak (1+) expressing cells or the microvessel density (number of vessels per mm^2) for each marker according to digital pathology analysis.
